# Electronic early notification of sepsis in hospitalized ward patients: a study protocol for a stepped-wedge cluster randomized controlled trial

**DOI:** 10.1186/s13063-021-05562-5

**Published:** 2021-10-11

**Authors:** Yaseen M. Arabi, Abdulmohsen Alsaawi, Mohammed Al Zahrani, Ali M. Al Khathaami, Raed H. AlHazme, Abdullah Al Mutrafy, Ali Al Qarni, Ahmed Al Shouabi, Eman Al Qasim, Sheryl Ann Abdukahil, Fawaz K. Al-Rabeah, Huda Al Ghamdi, Ebtisam Al Ghamdi, Mariam Alansari, Khadega A. Abuelgasim, Abdulaleem Alatassi, John Alchin, Hasan M. Al-Dorzi, Abdulaziz A. Ghamdi, Fahad Al-Hameed, Ahmad Alharbi, Mohamed Hussein, Wasil Jastaniah, Mufareh Edah AlKatheri, Hassan AlMarhabi, Hani T. Mustafa, Joan Jones, Saad Al-Qahtani, Shaher Qahtani, Ahmad S. Qureshi, Salih Bin Salih, Nahar Alselaim, Nabiha Tashkandi, Ramesh Kumar Vishwakarma, Emad AlWafi, Ali H. Alyami, Zeyad Alyousef, for the SCREEN Trial Group, Ahmed Al Arfaj, Mohamed S. Al Moammary, Soud Rasheed, Turki Alwasaidi, Amal Matroud, Rasha Ebeid Al Anazi, Amar M. Alhasani, Haifa Al Shammari, Majid M. Alshamrani, Saleh Qasim, Saeed Obbed, Adnan A. Munshi, Hadia Al Tabsh, Basem R. Banat, Omar Abuskout, Anna Liza Marcelo, Mayadah M. Alhabshi, Ibrahim J. Jaber, Mohammad Shahin, Jamielah Yaakob, Hanan Al Somali, Clara Masala, Mohammed Al Qarni, Jamal Chalabi, Johanna E. Greyvenstein, Abdul Rahman Jazieh, Noha Omaish, Azura Abdrahim, Mohammad Abdrabo, Abdullah Al Hamdan, Abdulaziz Al Qasem, Hattan Esilan

**Affiliations:** 1 College of Medicine, King Saud Bin Abdulaziz University for Health Sciences, King Abdullah International Medical Research Center, Intensive Care Department, King Abdulaziz Medical City, Ministry of National Guard Health Affairs, Riyadh, Saudi Arabia; 2 College of Medicine, King Saud Bin Abdulaziz University for Health Sciences, King Abdullah International Medical Research Center, King Abdulaziz Medical City, Ministry of National Guard Health Affairs, Riyadh, Saudi Arabia; 3 College of Medicine, King Saud Bin Abdulaziz University for Health Sciences, King Abdullah International Medical Research Center, King Abdulaziz Medical City, Ministry of National Guard Health Affairs, Jeddah, Saudi Arabia; 4 College of Medicine, King Saud Bin Abdulaziz University for Health Sciences, King Abdullah International Medical Research Center, Quality and Patient Safety Department, King Abdulaziz Medical City, Ministry of National Guard Health Affairs, Riyadh, Saudi Arabia; 5 College of Public Health and Health Informatics, King Saud Bin Abdulaziz University for Health Sciences, King Abdullah International Medical Research Center, Information Technology Department, King Abdulaziz Medical City, Ministry of National Guard Health Affairs, Riyadh, Saudi Arabia; 6 College of Osteopathic Medicine, Nova Southeastern University, Fort Lauderdale, Florida, USA; 7 College of Medicine, King Saud Bin Abdulaziz University for Health Sciences, King Abdullah International Medical Research Center, King Abdullah Specialized Children’s Hospital, Ministry of National Guard Health Affairs, Riyadh, Saudi Arabia; 8 Department of Medicine, King Abdulaziz Hospital, Ministry of National Guard Health Affairs, Al Ahsa, Saudi Arabia; 9 King Abdullah International Medical Research Center, Al Ahsa, Saudi Arabia; 10 King Saud bin Abdulaziz University for Health Sciences, Al Ahsa, Saudi Arabia; 11 Imam Abdulrahman Al Faisal Hospital, Ministry of National Guard Health Affairs, Dammam, Saudi Arabia; 12 College of Medicine, King Saud Bin Abdulaziz University for Health Sciences, Research Office, King Abdullah International Medical Research Center, Intensive Care Department, King Abdulaziz Medical City, Ministry of National Guard Health Affairs, Riyadh, Saudi Arabia; 13 Department of Internal Medicine, Imam Abdulrahman Al Faisal Hospital, Ministry of National Guard Health Affairs, Dammam, Saudi Arabia; 14 College of Medicine, King Saud Bin Abdulaziz University for Health Sciences, King Abdullah International Medical Research Center, Oncology Department, King Abdulaziz Medical City, Ministry of National Guard Health Affairs, Riyadh, Saudi Arabia; 15 King Saud Bin Abdulaziz University for Health Sciences, King Abdullah International Medical Research Center, Nursing Services Department, King Abdulaziz Medical City, Ministry of National Guard Health Affairs, Riyadh, Saudi Arabia; 16 College of Medicine, King Saud Bin Abdulaziz University for Health Sciences, King Abdullah International Medical Research Center, Quality and Patient Safety Department, King Abdulaziz Hospital Ministry of National Guard Health Affairs, Al Ahsa, Saudi Arabia; 17 College of Medicine, King Saud Bin Abdulaziz University for Health Sciences, King Abdullah International Medical Research Center, Intensive Care Department, King Abdulaziz Medical City, Ministry of National Guard Health Affairs, Jeddah, Saudi Arabia; 18 College of Medicine, King Saud Bin Abdulaziz University for Health Sciences, King Abdullah International Medical Research Center, Division of Infectious Diseases, Department of Medicine, King Abdulaziz Medical City, Ministry of National Guard Health Affairs, Riyadh, Saudi Arabia; 19 King Saud Bin Abdulaziz University for Health Sciences, Bioinformatics and Bioinformatics Department, King Abdullah International Medical Research Center, Ministry of National Guard Health Affairs, Riyadh, Saudi Arabia; 20 College of Medicine, King Saud Bin Abdulaziz University for Health Sciences, King Abdullah International Medical Research Center, Princess Noorah Oncology Center, King Abdulaziz Medical City, Ministry of National Guard Health Affairs, Jeddah, Saudi Arabia; 21 College of Medicine, King Saud Bin Abdulaziz University for Health Sciences, Quality and Patient Safety Department, Department of Medicine, King Abdulaziz Medical City, Ministry of National Guard Health Affairs, Jeddah, Saudi Arabia; 22 King Saud Bin Abdulaziz University for Health Sciences, King Abdullah International Medical Research Center, Quality and Patient Safety Department, Imam Abdulrahman Al Faisal Hospital, Ministry of National Guard Health Affairs, Dammam, Saudi Arabia; 23 College of Medicine, King Saud Bin Abdulaziz University for Health Sciences, King Abdullah International Medical Research Center, Intensive Care Department, Prince Mohammed bin Abdulaziz Hospital, Ministry of National Guard Health Affairs, Madinah, Saudi Arabia; 24 College of Medicine, King Saud Bin Abdulaziz University for Health Sciences, King Abdullah International Medical Research Center, Department of Medicine, King Abdulaziz Medical City, Ministry of National Guard Health Affairs, Riyadh, Saudi Arabia; 25 College of Medicine, King Saud Bin Abdulaziz University for Health Sciences, King Abdullah International Medical Research Center, Department of Surgery, King Abdulaziz Medical City, Ministry of National Guard Health Affairs, Riyadh, Saudi Arabia; 26 Saudi Nursing Professional Council, Saudi Commission for Health Specialties, Riyadh, Saudi Arabia; 27 College of Medicine, King Saud Bin Abdulaziz University for Health Sciences, King Abdullah International Medical Research Center, Department of Medicine, King Abdulaziz Medical City, Ministry of National Guard Health Affairs, Jeddah, Saudi Arabia; 28 College of Medicine, King Saud Bin Abdulaziz University for Health Sciences, King Abdullah International Medical Research Center, Department of Surgery, King Abdulaziz Medical City, Ministry of National Guard Health Affairs, Jeddah, Saudi Arabia

**Keywords:** Sepsis, Alert, Screening, qSOFA, Mortality, Electronic medical records

## Abstract

**Background:**

To evaluate the effect of screening for sepsis using an electronic sepsis alert vs. no alert in hospitalized ward patients on 90-day in-hospital mortality.

**Methods:**

The SCREEN trial is designed as a stepped-wedge cluster randomized controlled trial. Hospital wards (total of 45 wards, constituting clusters in this design) are randomized to have active alert vs. masked alert, 5 wards at a time, with each 5 wards constituting a sequence. The study consists of ten 2-month periods with a phased introduction of the intervention. In the first period, all wards have a masked alert for 2 months. Afterwards the intervention (alert system) is implemented in a new sequence every 2-month period until the intervention is implemented in all sequences. The intervention includes the implementation of an electronic alert system developed in the hospital electronic medical records based on the quick sequential organ failure assessment (qSOFA). The alert system sends notifications of “possible sepsis alert” to the bedside nurse, charge nurse, and primary medical team and requires an acknowledgment in the health information system from the bedside nurse and physician. The calculated sample size is 65,250. The primary endpoint is in-hospital mortality by 90 days.

**Discussion:**

The trial started on October 1, 2019, and is expected to complete patient follow-up by the end of October 2021.

**Trial registration:**

ClinicalTrials.gov NCT04078594. Registered on September 6, 2019

**Supplementary Information:**

The online version contains supplementary material available at 10.1186/s13063-021-05562-5.

## Background

Sepsis is a major cause of morbidity and mortality among hospitalized patients, and the outcome of affected patients is greatly dependent on the time-sensitive administration of appropriate antimicrobials, fluid resuscitation, and source control [1, 2]. Using machine learning analytics, a retrospective multicenter study evaluated the impact of delaying the implementation of the Surviving Sepsis Campaign 3-h bundle elements in 5072 adult patients with severe sepsis or septic shock [3]. The study found that the time delays significantly associated with mortality were 20 min for serum lactate, 50 min for blood culture, 100 min for intravenous crystalloids, and 125 min for antibiotic therapy [3]. Unfortunately, studies have shown that these elements of sepsis management are often delayed because of delayed sepsis recognition [4–6].

To facilitate early sepsis recognition, the Surviving Sepsis Campaign guidelines have recommended that hospitals have performance improvement programs for sepsis, including sepsis screening for acutely ill and high-risk patients [7]. Several observational studies have demonstrated that screening patients on hospital wards for sepsis was associated with improved processes of care and reduced mortality (please see also Supplemenrtary Introduction, Supplementary File). More recently, a randomized controlled trial (RCT) at two medical-surgical intensive care units (ICUs) compared an electronic sepsis alert with the same alert combined with a machine learning algorithm (experimental group) [8]. On receiving an alert, the care team evaluated the patient and initiated the severe sepsis bundle, if appropriate [8]. The length of stay decreased from 13.0 days in the control group to 10.3 days in the experimental group (*p* = 0.04) and hospital mortality by 12.4% (*p* = 0.02) [8]. A before-after study evaluated the impact of a multidisciplinary sepsis education program targeting sepsis recognition by using both the quick sequential organ failure assessment (qSOFA) and organ dysfunction criteria with nurse empowerment to activate the rapid response team (RRT) when sepsis was suspected [9]. The study found that the time to recognition (qSOFA-to-RRT) improved from a median of 11.8 h (interquartile range 3.4, 34.3) in the pre-intervention phase to 1.7 h (interquartile range 0, 11.7) in the post-intervention phase (*p* = 0.005) [9]. The time from qSOFA to antibiotics improved from 1.4 h (interquartile range:− 2.4, 6.2) to − 4.7 h (interquartile range − 25.4, 1.8, *p* < 0.01) [9]. Using qSOFA, compliance improved for antibiotics administration within 3 h from 60% to 87% (*p* = 0.02) [9]. A systematic review of 16 observational studies found that digital alert systems for sepsis were associated with a reduction in length of hospital stay [10].

On the other hand, some data questioned the benefit of sepsis screening among hospitalized patients. A systematic review of six studies of electronic and paper-based sepsis screening suggested improvement in processes of care including the use of diagnostics [11]. However, the effect on treatment (fluid resuscitation and antibiotic administration) and outcome measures was less consistent [11]. Most existing studies are pre-post intervention studies [6, 12–20], and showed large reductions in mortality even with modest improvement in the compliance with the bundle. One of the potential explanations is that early detection leads to the inclusion of mild cases (ascertainment bias) and therefore artificially improving mortality rates. Therefore, the observed effect may be exaggerated or even maybe solely related to this phenomenon. Two RCTs showed no benefit of screening for sepsis in the ICU setting. A trial in a medical ICU (*n* = 442) showed that electronic screening using modified systemic inflammatory response syndrome vs. usual care was not associated with a difference in time to new antibiotics, amount of fluid administered, ICU length of stay, hospital length of stay, and mortality [21]. Another trial in medical-surgical ICU patients (*n* = 417) found no difference between the electronic tool and usual care in the primary outcome of time to completion of all indicated Surviving Sepsis Campaign 6-h Sepsis Resuscitation Bundle elements, ICU mortality, ICU-free days, and ventilator-free days [22]. Besides, RCTs that tested real-time electronic alerts for clinical deterioration, which included sepsis in ward patients, found no significant difference in outcomes [23, 24]. Finally, screening for sepsis may lead to unintended consequences, such as overtreatment. One study found that the implementation of an electronic sepsis alert was associated with increase in antibiotic use and healthcare facility–onset *Clostridium difficile* infection [25]. Therefore, it is unclear whether screening for sepsis using an electronic alert in hospitalized patients improves outcomes, and an RCT is needed. The objective of the Stepped-wedge Cluster Randomized Controlled Trial of Electronic Early Notification of Sepsis in Hospitalized Ward Patients (SCREEN Trial) is to evaluate the effect of electronic screening for sepsis compared to no screening among hospitalized ward patients on all-cause 90-day in-hospital mortality. Our hypothesis is that screening for sepsis reduces all-cause 90-day in-hospital mortality.

## Methods

### Settings

The study is conducted in the 5 Ministry of National Guard Health Affairs (MNGHA) hospitals (King Abdulaziz Medical City - Riyadh, King Abdulaziz Medical City - Jeddah, and Prince Mohammed Bin Abdul Aziz Hospital - Al Madinah, King Abdulaziz Hospital - Al Ahsa, and Imam Abdulrahman Al Faisal Hospital - Dammam) (Supplementary file Table S1). The MNGHA hospitals share an integrated electronic medical record (EMR) system, BESTCare, which has been implemented as a joint venture with the Seoul National University Bundang Hospital, South Korea. Its critical applications include clinical documentation, computerized physician order entry, clinical decision support system, and clinical data warehouse. The system is also interfaced with the hospital’s vital signs measurement devices.

### Study design

**Fig. 1:**
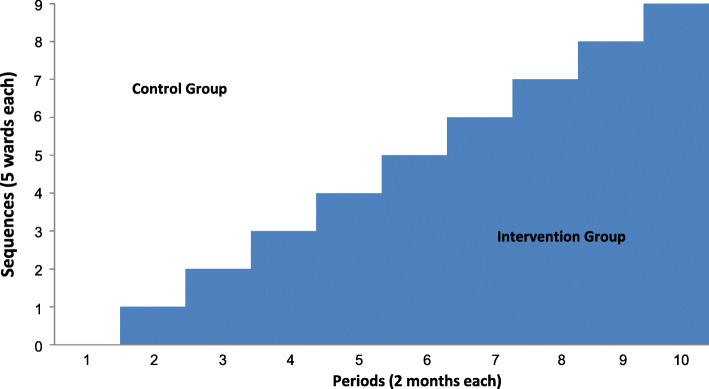
SCREEN trial design as a stepped-wedge cluster randomized trial

**Fig. 2:**
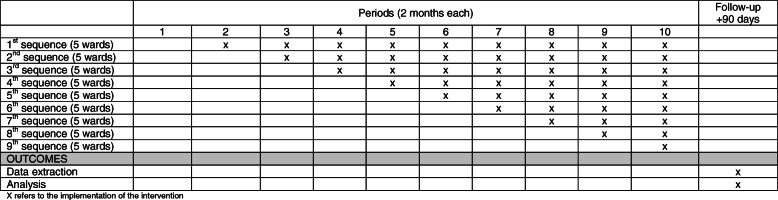
SPIRIT figure for the SCREEN trial

The SCREEN trial is designed as a stepped-wedge cluster RCT. Hospital wards (total of 45 wards, constituting clusters in this design) are randomized to have active alert vs. masked alert, 5 wards at a time, with every 5 wards constituting a sequence. The study consists of ten 2-month periods with a phased introduction of the intervention. In the first period, all wards had a masked alert for 2 months (see the “Sample size” section). After the baseline period, the intervention (alert system) is implemented in a new sequence every 2 months until the intervention is implemented in all sequences (Fig. 1). This design is suitable for quality improvement projects and incorporates the comparison between the intervention group and the control group, both horizontally (intervention clusters and control clusters at the same time) and vertically over time (the same cluster before and after the intervention). In addition, by having the masked alert, an additional comparison between patients with masked and active alerts will be performed. A SPIRIT figure is included in Fig. 2, with a checklist included as a Additional file 1.

This study was approved by the MNGHA Institutional Review Board (IRB). The study has been registered in ClinicalTrials.gov (NCT04078594).

### Inclusion and exclusion criteria

#### Ward level inclusion and exclusion criteria

Inclusion criterion

1. Inpatient wards, defined as wards used to manage inpatients, in the five MNGHA hospitals

Exclusion criteria

1. Cardiology, transplant, pediatric, and obstetric wards
2. ICUs and emergency departments
3. Operating rooms
4. Outpatient clinics
5. Daycare wards, endoscopy, outpatient procedure areas, and hemodialysis units

#### Patient-level inclusion and exclusion criteria

Inclusion criteria

1. Aged 14 years or older
2. Checked in as inpatient status to one of the study wards

Exclusion criteria

1. No commitment for full life support at the time of arrival to the study ward (designated as do-not-resuscitate status)

### Interventions

#### The sepsis alert

**Fig. 3:**
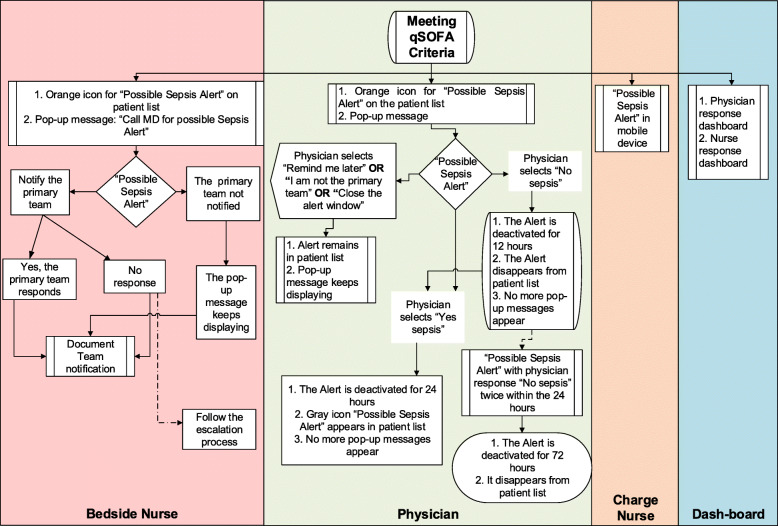
Workflow of the electronic alert

In the study wards, vital signs are logged into the electronic medical record (BESTCare system) every 4 h and the Glasgow Coma Scale (GCS) every 12 h. We developed a sepsis alert in the hospital information system based on qSOFA. The alert works as follows (Fig. 3 and Tables S2-S3, Supplementary File):

1. The screening tool automatically scans the documented vital signs and GCS and assigns one point for low blood pressure (systolic blood pressure ≤ 100 mmHg), increased respiratory rate (≥ 22 breaths/min), or altered mentation (Glasgow Coma Scale < 15).
2. If the total score is 2 or more based on the above criteria documented within 12 h, the system generates an electronic alert.
3. This alert results in the following:
  - In the bedside nurse workflow: The alert appears in the nurse worklist as “possible sepsis alert,” and a pop-up message appears requesting the nurse to notify the primary medical team and to document that a physician has received the message.
  - In the treating physician workflow: A pop-up message appears on the physician screen when the patient’s file is accessed as “possible sepsis alert.” The physician is requested to assess the patient condition and document whether the clinical condition is consistent with sepsis or not. Other options include “remind me later” and “I am not from the primary team.” By responding “yes,” the sepsis alert is closed and the system is deactivated for 24 h. By responding “no,” the system is deactivated for 12 h only. By responding “remind me later” or “I am not from the primary team,” the pop-up message appears on each subsequent physician access of the file (Fig. 3).
  - The ward charge nurse gets a notification on a mobile device (iPod) that has a custom-built application for an immediate alert with visual and sound notification.
  - If the goals of care for a given patient are modified to do-not-resuscitate, the system is deactivated for that patient.
4. Data are also used to create a dashboard for each ward with the following indicators:
  - Number of alerts.
  - Percent of alerts with documented notification by the nurse to the primary medical team and time to notification.
  - Percent of alerts with documented assessment by the physician and time to assessment.
  - Access to the dashboard is provided to all nursing and physician managers. Reports and feedback are provided every 2 weeks, with frequent meetings to review the results and discuss the approaches for improvement in the involved wards.

#### Intervention group (active alert)

In this group of wards, the alert system is active, which means that the alerts are visible to the treating team as described above. The alert prompts a nurse-to-physician communication and a physician assessment of patients.

#### Control group (masked alert)

In this group of wards, the alert system will be inactive in the EMR frontend for the treating team and will remain active in the backend of the EMR. Sepsis recognition is according to the existing practice. This enables collecting data on the sepsis alert patients whether they are on active or masked control wards.

#### Co-interventions

A hospital-wide sepsis awareness campaign in all 5 hospitals was started at the beginning of the study with training sessions open to all staff on the importance of timely interventions for sepsis. Project in-service training sessions are provided to the involved medical and nursing departments at the beginning of each period. Monthly webinars are conducted with active ward leaders. An intranet page has been developed with educational resources (videos, presentations, documents, posters, and related links) that explain this project and provide guidance. The medical management and assessment are at the discretion of the treating team. However, this project adopts the 2016 Surviving Sepsis Campaign guidelines and the hour 1 bundle, but without any specific monitoring of bundle compliance [7, 26, 27].

### Randomization

The five hospitals have collectively 46 eligible wards; two wards were merged due to similarities in order to create 9 sequences of 5 wards each. Wards are randomized to receive the intervention as a 5-ward sequence each 2-month period according to a computer-generated non-stratified concealed list. The randomization list was maintained with a research coordinator who was not involved in this trial, and the ward allocation remained concealed from the research and clinical teams throughout the study and was revealed for a given sequence only 1 month before the implementation of the intervention to allow training (Fig. 1).

### Data

#### Data extraction

All data will be extracted from the EMR. Data will be extracted as per the definitions in Table 1 and Supplementary file Tables S4-S10.

**Table 1:** Time windows for data extraction. All data points within the time window will be extracted

Variables	Attribute	Type of variable	ITT cohort	Alert cohort
Age (years)				
Sex				
Location before ward check-in	All values			
Service	All values			
Admission diagnoses, main and secondary	All ICD-10-AM^a^			
Problem list	All ICD-10-AM^a^			
Discharge diagnosis	All ICD-10-AM^a^			
Alert criteria		Baseline data		
Alert count	All alert dates and times	Baseline data		
Time to acknowledgment by nurse and physician	First alert: time to acknowledgment by nurse and by physician	Process measure		
Vital signs (heart rate (HR), systolic blood pressure (SBP), diastolic blood pressure (DBP), respiratory rate (RR), temperature)	All values, dates and times	Baseline data	Check-in to + 12 h	− 12 h to alert time
The same vital signs except for temperature	All values, dates and times	Physiologic outcome		Alert time to + 12 h
Lactate	All values, dates and times	Baseline data	− 12 to + 12 h	− 12 h to alert time
Lactate	All values, dates and times	Process measure, physiologic outcome		Alert time to + 12 h
Other blood tests results (white blood cell count, bilirubin, creatinine)	All values, dates and times	Baseline data	− 12 to + 12 h	− 12 h to alert time
Cultures obtained (blood, respiratory, urine, body fluid ((pleural, ascitic, CSF, joint))	Yes vs. no, dates and times, results	Baseline data	− 12 to + 12 h	− 12 h to alert time
Cultures obtained (blood, respiratory, urine, body fluid ((pleural, ascitic, CSF, joint))	Yes vs. no, dates and times, results	Process measure		Alert time to + 12 h
Intravenous fluids administration prescribed	Yes vs. no, date and time	Baseline data	− 12 to + 12 h	− 12 h to alert time
	Yes vs. no, date and times	Process measure		Alert to + 12 h
Hemodialysis	Yes vs. no	Baseline data	Dialysis (hemodialysis) before check-in as outpatient or as in patient in the week preceding check-in	Dialysis (hemodialysis) before alert as outpatient or as in patient in the week preceding check-in
	Yes vs. no, first date and time only	Outcome	Hemodialysis post check-in during this hospital stay (including during ICU stay)	Dialysis within 14 days from the alert (including during ICU stay)
Continuous renal replacement therapy (CRRT)	Yes vs. no, first date and time only	Outcome	CRRT post-check-in during this hospital stay (including during ICU stay)	CRRT within 14 days from the alert (including during ICU stay)
Vasopressors (norepinephrine, dopamine, epinephrine, dobutamine, phenylephrine, vasopressin)	Yes vs. no, date and time of initiation	Outcome	Check-in to 90 days	Alert to 14 days
Mechanical ventilation (except operation room)	Yes vs. no, date and time of initiation	Outcome	Check-in to 90 days	Alert to 14 days
ICU admission	Yes vs. no, date and time of initiation, number of days in ICU	Outcome	Check-in to 90 days	Alert to 14 days
Critical care rapid response team (CCRT) activation	Yes vs. no, date and time	Outcome	Check-in to 90 days	Alert to 14 days
Code blue	Yes vs. no, date and time	Outcome	Check-in to 90 days	Alert to 14 days
Hospital discharge	Date and time, status	Outcome		
Antibiotics	Yes vs. No, date and time^b^	Baseline data	− 12 to + 12 h	− 12 h to alert time
		Process measure		Alert to + 12 h
	Yes vs. no, number of days on antibiotics	Outcome	Check-in to 90 days	Alert to 14 days
Multidrug-resistant organism (MDROs)	Yes vs. no, type, date and time	Outcome	Check-in to 90 days	Alert to 90 days
*Clostridium difficile* (C-diff)	Yes vs. no, date and time	Outcome	Check-in to 90 days	Alert to 90 days

^a^ICD-10-AM: diagnosis codes used for comorbidity and for infections are referenced in the supplementary file (Table S4 & S5)

^b^Antibiotics list is referenced in the supplementary file (Table S6)

#### Baseline data, physiological parameters and treatments at baseline, and processes measures

These variables are listed in the Supplementary Methods and Tables S8 and S9.

### Outcomes

#### Primary outcome (Supplementary file Table S10)

- All-cause in-hospital mortality within 90 days

#### Secondary outcomes

*Outcome measures*

- Hospital length of stay (LOS), censored at 90 days
- Transfer to ICU within 90 days (ITT cohort) and 14 days of alert (alert cohort)
- ICU-free days in the first 90 days (ITT cohort and alert cohort)
- Critical Care Response Team (CCRT) activation within 90 days (ITT cohort) and 14 days of alert (alert cohort)
- Cardiac arrest within 90 days (ITT cohort) and 14 days of alert (alert cohort)
- The need for mechanical ventilation, vasopressor therapy, and incident renal replacement therapy within 90 days (ITT cohort) and 14 days of alert (alert cohort)

*Balancing measures/safety outcomes*

- Antibiotic-free days within 90 days (ITT cohort and alert cohort)
- The acquisition of multidrug-resistant organisms within 90 days (ITT cohort and alert cohort)
- *Clostridium difficile* infection within 90 days (ITT cohort and alert cohort)

### Sample size

The sample size for this stepped-wedge cluster-randomized design was calculated for 45 clusters, each 5 clusters constitute a sequence with 10 periods (including one baseline period) using the Power Analysis and Sample Size (PASS) software (PASS 15 Power Analysis and Sample Size Software (2017), NCSS, LLC. Kaysville, UT, USA, ncss.com/software/pass). After a baseline period, a new sequence of 5 clusters (wards) switch from the control group to the intervention group at the beginning of each subsequent period. Using historical data obtained from the development domain of the EMR for ward patients admitted from 01 July 2018 to 30 June 2019, we calculated a baseline in-hospital mortality rate by day 90 of 3.13%. Based on the same dataset, 18.3% of eligible ward patients had an alert based on the qSOFA criteria [28] with an in-hospital mortality of 8.16% compared to 2% in the patients with no qSOFA. For sample size calculations, we made the following assumptions: (A) the impact of the intervention on mortality occurs only in patients who have the alert, (B) only half of the patients with the alert have sepsis, (C) 90% of deaths among the patients with the alert occurred among septic patients, (D) early intervention resulting from the alert will reduce the in-hospital mortality by 50%, i.e., from 8.16 to 4.08% in patients with sepsis and would lead to an overall change in in-hospital mortality for the whole cohort from 3.13 to 2.46%, (E) 80% power using two-sided Wald *Z*-test and significance level of 5%, and (F) an intra-cluster correlation (ICC, a measure of the relatedness of cluster) of 0.22 as estimated from the same retrospective electronic database. As the primary analysis would be adjusted for the random effect to account for the correlation between patients within the same cluster, we used the estimation variance (*σ*^2^), which was calculated from responses (P1, P2), as the within-cluster variance (*σw*^2^) as suggested by Hussey and Hughes (2007) and Hemming and Girling (2014) [29, 30]. As such, a reduction of in-mortality in hospital by 90 days by 0.67% (from 3.13 to 2.46%) requires a total sample size of 65,250 subjects (average of 1450 subjects per cluster with an average of 145 subjects per cluster per period). With all five hospitals combined, this is expected to require 20 months (2 months per period).

### Statistical analysis

For the SCREEN trial, the analysis will be implemented under the guiding principles of the ICH E9 for the analysis of randomized controlled trials [31]. The stepped-wedge design is essentially a matched design with before and after comparisons for each unit of randomization (or cluster), which is the ward in this study (additional information is in the Supplementary Methods)

### Study cohorts

#### Intention-to-treat cohort

We will report patient flow according to the CONSORT flowchart for the stepped-wedge cluster-randomized trial by allocated sequence and period. The intention-to-treat (ITT) cohort includes all eligible patients admitted to the eligible wards. The ITT analysis also implies that patients in the ITT cohort in the wards belonging to a particular period will be analyzed in accordance with their planned randomization regardless of what happens during the trial. For example, if a ward was planned to have the intervention during a given period and for technical reasons that alert system was not operational, patients in that ward during that period will be analyzed as receiving an active alert. Although it is not anticipated that there will be wards that cross over their study cohort (i.e., change from alert to non-alert or vice versa), any such instances will be documented. Patients who are transferred from one ward to another will be counted as part of the first ward. The primary analysis will be based on this population.

The study was launched in October 2019. During the study period in 2020–2021, and in response to the surge in the number of hospitalized patients with coronavirus disease 2019 (COVID-19) [32], some of the wards had to be converted to ICUs, making them ineligible for the study intervention. These wards will be excluded from the ITT cohort while used as ICUs. During the peak of COVID-19 cases, total admissions to the wards declined substantially to less than 50%; therefore, 2 consecutive periods (starting June 2019) were extended from 2 to 3 months each to account for the decline in cluster size. In addition, some wards were designated for admission of suspected or confirmed COVID-19 cases. Given the higher mortality associated with such designation, and because such designation would likely be over-represented in the intervention group, data on a ward designated as a COVID-19 ward will be documented as a ward-level variable for inclusion as an adjustment in the primary model (as discussed below). Additionally, patient-level data on COVID-19 diagnosis will be also obtained.

### Alert cohort

This cohort represents the subset of ITT patients who have the alert whether in the intervention wards or the control wards.

### Analysis of the primary endpoint

The analysis for the primary outcome will be conducted on the ITT cohort. The all-cause in-hospital mortality by day 90 will be compared between the intervention group and the control group at the individual level with the use of a mixed-effect logistic regression model with the jack-knife method to estimate standard errors to account for grouping within clusters [29, 33]. We will use a generalized linear mixed model with a binary distribution and a log-link function to estimate the relative risk as a measure of effect. We will include two levels of random effects to account for nested clustering within wards and periods and two levels of fixed effects: hospitals and COVID-19 ward status. The model will be selected as the best model with a unique covariance structure that produces the lowest Bayesian Information Criterion (BIC) value. The covariance structures that will be considered in the model are the first order of autocorrelation covariance structure, unstructured covariance structure, Toeplitz covariance structure, and variance component structure (VC). The random coefficients will be modeled using G-side random effects and the subject-specific estimates will be obtained by defining the appropriate variance-covariance structure.

### Analysis of other outcomes

The categorical outcomes will be summarized between the intervention and control groups using number and percentages and analyzed in a similar manner as done for the primary outcome. The continuous outcomes will be summarized using mean and standard deviation or median and inter-quartile ranges (IQR) and will be analyzed using the mixed-effect Poisson model. We will include two levels of random effects to account for nested clustering within wards and periods and two levels of fixed effects: hospitals and COVID-19 ward status.

### Subgroups analysis

To address the concern about contamination, we will conduct a sensitivity analysis excluding all patients in the control group in the 90 days before crossing over to the intervention group. Because there are fewer than 50 clusters, we will conduct a sensitivity analysis using a small sample correction with a Kenward-Roger method [34]. We will conduct a sensitivity analysis adjusting for the following covariates: type of wards (medical, surgical, oncology, and mixed), age, baseline systolic blood pressure, baseline respiratory rate, GCS, Charlson comorbidity index, and COVID-19 status. For the latter analysis, we will use imputation for missing variables. In addition, we will conduct also a complete case sensitivity analysis. We will conduct a sensitivity analysis excluding the periods in which wards were assigned as COVID-19 ward.

We will analyze the primary outcome of all-cause hospital mortality by day 90 across predefined subgroups using the same model of the primary analysis. The predefined subgroups include age ≤ 65 years and > 65 years; patients with documented infection source (including ICD-10 AM for pneumonia, urinary tract infection, skin and soft tissue infection, intra-abdominal infection, or other infections); patients with no documented infection or infection source; patients admitted to medical, surgical, oncology, and mixed wards; alert within 48 h of admission and after 48 h of admission; and patients admitted to COVID-19 and non-COVID-19 wards (Table S6). The results of the test of interaction will be reported.

All analyses will be conducted with SAS version 9.4. There will be no interim analysis due to the nature of implementation.

## Discussion

This trial evaluates the value of electronic screening for sepsis in patients hospitalized in the wards. Screening for sepsis could be of substantial benefit as sepsis is frequent in hospitalized patients, is often under-recognized, and is a common cause of organ dysfunction and death, and earlier appropriate management is associated with improved outcomes [3–6]. It is estimated that 32% of sepsis cases are identified in hospital wards [35], such that hospital-onset sepsis complicates 1 in 200 hospitalizations with an associated mortality of > 30% [36, 37]. Sepsis management is often delayed among patients hospitalized in the ward [38, 39]. A study found that lactate levels were measured within the mandated window in only 32% of patients with severe sepsis in the ward compared with 55% in the ICU and 79% in the ED [39]. Delayed lactate level measurement was associated with longer time to antibiotic administration [39]. The evidence for electronic alerts to recognize sepsis early and improve care processes and outcomes in general ward patients is modest [40], which affirms the need for an RCT. The stepped-wedge approach to include wards in this trial avoids several pitfalls and retains controlled data elements and randomization.

Real-time electronic screening for sepsis using the EMR provides multiple advantages over other methods, as it is considered a low-cost solution for more reliable, reproducible, unbiased, and sustainable screening in large hospitals [41]. However, the challenges encountered with our electronic alert development and implementation included resource allocation, changing and unagreed-upon sepsis screening tools (qSOFA and SIRS), charting behaviors, alert fatigue (false positive), inappropriate response (false negative), and differences in health care delivery models [42, 43]. We have conducted multiple presentations to ward medical and nursing staff and provided online materials on the alert system and sepsis management in line with the Surviving Sepsis Campaign guidelines [7, 26]. The actual management of patients with positive sepsis alert was left to the discretion of the treating team. We also utilized dashboards during the study to provide opportunities for ward medical and nursing leaders to review and improve provided care.

The SCREEN trial provides an opportunity for a novel trial design and analysis of routinely collected and entered data to evaluate the effectiveness of an intervention (sepsis alert) for a common medical problem (sepsis in ward patients). Its results may open the door for other trials on other interventions in other conditions.

## Trial status

The trial started on October 1, 2019, and is expected to complete patient follow-up by the end of October 2021. The study was conducted according to the protocol in its 3rd version dated September 15, 2019. There have been minor protocol modifications after starting the trial. The first was extending two periods because of the COVID-19 pandemic as described above, which was communicated at the time with involved wards. An additional amendment was made in August 2021 in response to the peer-review process of the statistical analysis plan. The amendment included a few clarifications on the study outcomes and a change in the approach to calculate the sample size resulting in the change in the sample size from 62,550 to 65,250. These changes have no impact on the study conduct.

## Supplementary Information

**Additional file 1.** SPIRIT 2013 Checklist: Recommended items to address in a clinical trial protocol and related documents.

**Additional file 2.** Supplementary file.

## Data Availability

The datasets will be available from the corresponding author as per the regulations of KAIMRC.

## Abbreviations

CCRT: Critical Care Response Team
ED: Emergency department
EMR: Electronic medical record
ICU: Intensive care unit
ITT: Intention-to-treat
LOS: Length of stay
qSOFA: Quick sequential organ failure assessment
SIRS: Systemic inflammatory response syndrome
SOFA: Sequential organ failure assessment
