# Supplementary material for: Electronic early notification of sepsis in hospitalized ward patients: a study protocol for a stepped-wedge cluster randomized controlled trial
Source: Trials. 2021 Oct 11;22:695. doi: 10.1186/s13063-021-05562-5 (PMC8503718; doi:10.1186/s13063-021-05562-5)
Supplement: Supplementary file 1 — Additional file 1. SPIRIT 2013 Checklist: Recommended items to address in a clinical trial protocol and related documents. [file 13063_2021_5562_MOESM1_ESM.docx]

Supplementary File page 4-5

26

26

26


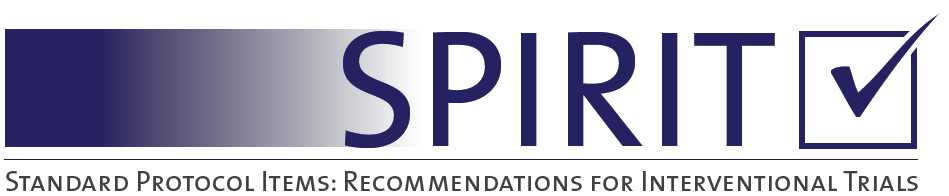


SPIRIT 2013 Checklist: Recommended items to address in a clinical trial protocol and related documents*

**Section/item**

**Item No**

**Description**

**Addressed on page number**

**Administrative information**

Title

1

Descriptive title identifying the study design, population, interventions, and, if applicable, trial acronym

Trial registration

2a

Trial identifier and registry name. If not yet registered, name of intended registry

2b

All items from the World Health Organization Trial Registration Data Set

Protocol version

3

Date and version identifier

Funding

4

Sources and types of financial, material, and other support

Roles and

responsibilities

5a

Names, affiliations, and roles of protocol contributors

5b

Name and contact information for the trial sponsor

5c

Role of study sponsor and funders, if any, in study design; collection, management, analysis, and

interpretation of data; writing of the report; and the decision to submit the report for publication, including whether they will have ultimate authority over any of these activities

5d

Composition, roles, and responsibilities of the coordinating centre, steering committee, endpoint adjudication committee, data management team, and other individuals or groups overseeing the trial, if

applicable (see Item 21a for data monitoring committee)

1

1,9-10

8

8

25

1-6 ,27-30

**Introduction**

Background and

rationale

6a

Description of research question and justification for undertaking the trial, including summary of relevant

studies (published and unpublished) examining benefits and harms for each intervention

6b

Explanation for choice of comparators

Objectives

7

Specific objectives or hypotheses

Trial design

8

Description of trial design including type of trial (eg, parallel group, crossover, factorial, single group),

allocation ratio, and framework (eg, superiority, equivalence, noninferiority, exploratory)

**Methods: Participants, interventions, and outcomes**

Study setting

9

Description of study settings (eg, community clinic, academic hospital) and list of countries where data will

be collected. Reference to where list of study sites can be obtained

Eligibility criteria

10

Inclusion and exclusion criteria for participants. If applicable, eligibility criteria for study centres and

individuals who will perform the interventions (eg, surgeons, psychotherapists)

Interventions

11a

Interventions for each group with sufficient detail to allow replication, including how and when they will be

administered

11b

Criteria for discontinuing or modifying allocated interventions for a given trial participant (eg, drug dose

change in response to harms, participant request, or improving/worsening disease)

11c

Strategies to improve adherence to intervention protocols, and any procedures for monitoring adherence

(eg, drug tablet return, laboratory tests)

11d

Relevant concomitant care and interventions that are permitted or prohibited during the trial

Outcomes

12

Primary, secondary, and other outcomes, including the specific measurement variable (eg, systolic blood pressure), analysis metric (eg, change from baseline, final value, time to event), method of aggregation (eg, median, proportion), and time point for each outcome. Explanation of the clinical relevance of chosen

efficacy and harm outcomes is strongly recommended

Participant timeline

13

Time schedule of enrolment, interventions (including any run-ins and washouts), assessments, and visits for

participants. A schematic diagram is highly recommended (see Figure)

2

9-11

11-12

11

11-12

11

13-14

13-15

14-16

14-16

16

18

12-13

19

Sample size

14

Estimated number of participants needed to achieve study objectives and how it was determined, including

clinical and statistical assumptions supporting any sample size calculations

Recruitment

15

Strategies for achieving adequate participant enrolment to reach target sample size

**Methods: Assignment of interventions (for controlled trials)**

Allocation:

Sequence

generation

16a

Method of generating the allocation sequence (eg, computer-generated random numbers), and list of any factors for stratification. To reduce predictability of a random sequence, details of any planned restriction (eg, blocking) should be provided in a separate document that is unavailable to those who enrol participants

or assign interventions

Allocation concealment

mechanism

16b

Mechanism of implementing the allocation sequence (eg, central telephone; sequentially numbered,

opaque, sealed envelopes), describing any steps to conceal the sequence until interventions are assigned

Implementation

16c

Who will generate the allocation sequence, who will enrol participants, and who will assign participants to

interventions

Blinding (masking)

17a

Who will be blinded after assignment to interventions (eg, trial participants, care providers, outcome

assessors, data analysts), and how

17b

If blinded, circumstances under which unblinding is permissible, and procedure for revealing a participant’s

allocated intervention during the trial

**Methods: Data collection, management, and analysis**

Data collection

methods

18a

Plans for assessment and collection of outcome, baseline, and other trial data, including any related processes to promote data quality (eg, duplicate measurements, training of assessors) and a description of study instruments (eg, questionnaires, laboratory tests) along with their reliability and validity, if known.

Reference to where data collection forms can be found, if not in the protocol

18b

Plans to promote participant retention and complete follow-up, including list of any outcome data to be

collected for participants who discontinue or deviate from intervention protocols

3

19

16-17

16-17

16-17

16-17

16-17

17-18

17-18

Supplementary File page 4-5

Supplementary File page 4-5

Supplementary File page 4

19

Supplementary File page 4-5;

Supplementary File page 4-5

16-19

Data management

19

Plans for data entry, coding, security, and storage, including any related processes to promote data quality (eg, double data entry; range checks for data values). Reference to where details of data management

procedures can be found, if not in the protocol

Statistical methods

20a

Statistical methods for analysing primary and secondary outcomes. Reference to where other details of the

statistical analysis plan can be found, if not in the protocol

20b

Methods for any additional analyses (eg, subgroup and adjusted analyses)

20c

Definition of analysis population relating to protocol non-adherence (eg, as randomised analysis), and any

statistical methods to handle missing data (eg, multiple imputation)

**Methods: Monitoring**

Data monitoring

21a

Composition of data monitoring committee (DMC); summary of its role and reporting structure; statement of whether it is independent from the sponsor and competing interests; and reference to where further details about its charter can be found, if not in the protocol. Alternatively, an explanation of why a DMC is not

needed

21b

Description of any interim analyses and stopping guidelines, including who will have access to these interim

results and make the final decision to terminate the trial

Harms

22

Plans for collecting, assessing, reporting, and managing solicited and spontaneously reported adverse

events and other unintended effects of trial interventions or trial conduct

Auditing

23

Frequency and procedures for auditing trial conduct, if any, and whether the process will be independent

from investigators and the sponsor

**Ethics and dissemination**

Research ethics

approval

24

Plans for seeking research ethics committee/institutional review board (REC/IRB) approval

Protocol

amendments

25

Plans for communicating important protocol modifications (eg, changes to eligibility criteria, outcomes,

analyses) to relevant parties (eg, investigators, REC/IRBs, trial participants, trial registries, journals, regulators)

4

18

18

17

24

Supplementary File page 4-5, 24

Supplementary File page 4-5

Supplementary File page 4-5

N/A; the protocol manuscript was written by the authors

N/A, no samples will be collected

N/A, no will be samples collected

Consent or assent

26a

Who will obtain informed consent or assent from potential trial participants or authorised surrogates, and

how (see Item 32)

26b

Additional consent provisions for collection and use of participant data and biological specimens in ancillary

studies, if applicable

Confidentiality

27

How personal information about potential and enrolled participants will be collected, shared, and maintained

in order to protect confidentiality before, during, and after the trial

Declaration of

interests

28

Financial and other competing interests for principal investigators for the overall trial and each study site

Access to data

29

Statement of who will have access to the final trial dataset, and disclosure of contractual agreements that

limit such access for investigators

Ancillary and post-

trial care

30

Provisions, if any, for ancillary and post-trial care, and for compensation to those who suffer harm from trial

participation

Dissemination policy

31a

Plans for investigators and sponsor to communicate trial results to participants, healthcare professionals,

the public, and other relevant groups (eg, via publication, reporting in results databases, or other data sharing arrangements), including any publication restrictions

31b

Authorship eligibility guidelines and any intended use of professional writers

31c

Plans, if any, for granting public access to the full protocol, participant-level dataset, and statistical code

**Appendices**

Informed consent

materials

32

Model consent form and other related documentation given to participants and authorised surrogates

Biological

specimens

33

Plans for collection, laboratory evaluation, and storage of biological specimens for genetic or molecular

analysis in the current trial and for future use in ancillary studies, if applicable

*It is strongly recommended that this checklist be read in conjunction with the SPIRIT 2013 Explanation & Elaboration for important clarification on the items. Amendments to the protocol should be tracked and dated. The SPIRIT checklist is copyrighted by the SPIRIT Group under the Creative Commons

“[Attribution-NonCommercial-NoDerivs 3.0 Unported](http://www.creativecommons.org/licenses/by-nc-nd/3.0/)” license.

5

24

22-23

26

26

24
