# Supplementary material for: Electronic early notification of sepsis in hospitalized ward patients: a study protocol for a stepped-wedge cluster randomized controlled trial
Source: Trials. 2021 Oct 11;22:695. doi: 10.1186/s13063-021-05562-5 (PMC8503718; doi:10.1186/s13063-021-05562-5)
Supplement: Supplementary file 2 — Additional file 2. Supplementary file. [file 13063_2021_5562_MOESM2_ESM.pdf]

Supplementary Introduction

A study from our center found that the implementation of a multifaceted intervention including sepsis electronic‑alert (e-alert) with sepsis response team was associated with earlier identification of sepsis, increase in compliance with sepsis resuscitation bundle, and reduction in the need for mechanical ventilation and in-hospital mortality and length of stay.[1] A pre and post study evaluated a system using a triad of real-time electronic surveillance, mobile alerts received by nurses for all positive sepsis screenings as well as severe sepsis and shock alerts, and specific decision support for early goal-directed therapy.[2] During the study period, sepsis-related mortality decreased by 53% from 90 to 42 deaths per 1000 sepsis cases (p=0.03) and the 30-day readmission rate by 30.8% from 19.1% to 13.2% (p=0.05).[2] A prospective observational study compared a real-time sepsis alert with no alert in 6 medical wards in a 1250-bed academic medical center.[3] The study found that within 12 hours of the sepsis alert, 70.8% of patients in the intervention group had received ≥1 intervention vs. 55.8% in the control group (p=0.02).[3] Antibiotic escalation, intravenous fluid administration, oxygen therapy, and diagnostic tests were all increased in the intervention group.[3]

Supplementary Methods

Baseline Data (ITT and alert cohorts- Supplementary file Table S7)

- Age and Sex
- Admission source: emergency room, operating room, clinic, ICU, others
- Admitting ward: medical, surgical, oncology, mixed (any combination)
- Co morbidities: end-stage renal disease, cancer without metastasis, immune-compromised non-complicated, diabetes, complicated diabetes, congestive heart failure, acquired immunodeficiency syndrome, moderate to severe chronic kidney disease, myocardial infarction, chronic pulmonary disease, peripheral vascular disease, stroke or

transient ischemic attack, dementia, hemiplegia or paraplegia, connective tissue disease, peptic ulcer disease, mild liver disease, moderate to severe liver disease

- Charlson Comorbidity Index
- Source of infection on admission to the ward: No infection, pneumonia, urinary tract infection, skin and soft tissue infection, intra-abdominal infection, other infections
- Dialysis
- Alert count, time to first alert and the parameters leading to alert (respiratory rate ≥22 breath/minute, systolic blood pressure ≤100 mmHg, GCS <15)

Physiological parameters and treatments at baseline (ITT population) and pre-alert (Alert population) (Supplementary file Table S8)

- Systolic and diastolic blood pressure: lowest values in the first 12 hours of check in to the ward (ITT population) and in the 12 hours before the alert
- Heart rate and respiratory rate: highest values in the first 12 hours of check in to the ward (ITT population) and in the 12 hours before the alert
- Temperature: highest value and lowest value in the first 12 hours of check in to the ward (ITT population) and in the 12 hours before the alert
- Lactate level: highest lactate 12 hours before check in to the ward to 12 hours of check-in to the ward (ITT population) and highest value in the 12 hours before the alert (alert population).
- White blood cell count, bilirubin, and creatinine: highest values in the 12 hours before check-in to the ward to 12 hours after check-in to the ward (ITT population) and highest value in the 12 hours before alert (alert population)
- Blood, respiratory, urine and body fluid cultures: percentage of patients with cultures ordered in the 12 hours before check-in to the ward to 12 hours after check-in to the ward (ITT population) and in the 12 hours before alert (alert population)

- Intravenous fluid (NS, ½NS, D5NS, D5½NS, LR, D5LR, albumin 5%, 20%) given in the 12 hours before check-in to the ward to 12 hours after check-in to the ward (ITT population) and in the 12 hours before the alert (alert population)
- Antibiotics: percentage of patients on antibiotics in the 12 hours before check-in to the ward to 12 hours after check-in to the ward (ITT population) and in the 12 hours before alert (alert population)

Processes measures (Supplementary file Table S9)

- Post-alert lactate level
  - Percentage of patients with lactate reported 12 hours if not reported in the 12 hours before alert
  - Highest value reported in the 12 hours after the alert
- Post-alert blood culture:
  - Percentage of patients with blood culture ordered in 12 hours if not performed in the 12 hours before alert
- Post-alert respiratory, urine and body fluid cultures
  - Percentage of patients with respiratory, urine and body fluid cultures ordered in 12 hours if not performed in the 12 hours before alert
  - Intravenous fluid administered in 12 hours after alert (yes, no)
- Post-alert antibiotics
  - Percentage of patients who were not on antibiotics in the 12 hours before alert and had new antibiotic administered within 3 and 12 hours of alert
  - Percentage of patients who were on antibiotics in the 12 hours before alert and had new antibiotic administered within 3 and 12 hours of alert
- Post-alert systolic blood pressure: lowest value in the 12 hours after the alert.
- Post-alert diastolic blood pressure: lowest value in the 12 hours after the alert
- Post-alert heart rate: highest value in the 12 hours after the alert
- Post-alert respiratory rate: highest value respiratory in the 12 hours after the alert

Supplementary Statistical analysis

Raw data will be processed in accordance with the best practices for raw data management to identify any inaccuracies or incompleteness in advance of the statistical analysis. In order to accomplish this task, all variables will be checked and summarized in terms of maximum and minimum values. Minimum and maximum values will be checked and compared against the nominal maximum and minimum value of each variable, and variables with implausible values will be flagged. All variables will be summarized and reported for the study using descriptive statistics. Variables will be summarized and reported in terms of n, mean and median, standard deviation, the first and third quartiles. Categorical variables will be summarized and reported in terms of frequency distribution. All demographic and clinical variables would be summarized between study groups at baseline by wards and overall.

Ethical considerations

This is a quality improvement project that is implemented in the participating hospitals in phases. The stepped wedge design is a well-accepted for this type of project,[4] since it ensures that all patients will eventually receive an intervention that is considered to be beneficial. It is expected that the quality of care might improve with time by using the electronic sepsis alert and detecting all at high risk patients. Moreover, the study does not interfere with the routine management of patients and does not require any direct interaction between the research team and patients.[5]. Additionally, data are obtained from the electronic medical records for a large sample size of ward patients. Therefore, this study is considered as minimal risk and consent is not required, similar to other studies of the same nature.[6, 7]. In accordance with Good Clinical Practice guidelines, there will be no patient identifying information will be used in any publication or be shared with other parties.

Study Governance

The Steering Committee members will be responsible for overseeing the conduct of the trial, for upholding or modifying study procedures as needed, addressing challenges with protocol implementation, formulating the analysis plan, reviewing and interpreting the data, and preparing the manuscript. All study-related data files are kept in password-protected computers with restricted access. Given the nature of the trial, there will be ongoing audit and feedback to the participating wards about the performance, but audit for data collection is no applicable. Upon completion, the results of the trial is planned to be published in peer-reviewed journal and be shared with public.

Supplementary Discussion:

The optimal tool for screening for sepsis has been debated. A qSOFA score ≥ 2 has been suggested for identification of patients at risk of sepsis outside the ICU. A study in medical wards (481 patients) found that qSOFA had lower sensitivity (44.7% vs. 80.0%), but higher specificity (83.6% vs. 25.7%) and positive predictive value (75.5% vs. 54.8%) for predicting sepsis compared with SIRS.[8] A large prospective cohort study of approximately 1 million hospitalized patients in 85 US hospitals found that 27.0% were qSOFA-positive within 1 day of admission.[9] The sensitivities of qSOFA for suspected infection and sepsis were 41% and 63%, respectively and the positive predictive values were 31% and 17%, respectively.[9] As we prepared for this trial, we initially used SIRS to build the electronic screening tool. Based on analysis of historical data from our hospital information system, qSOFA identified ward patients who were at risk for subsequent ICU admission and death more frequently and earlier than SIRS.[10] Therefore, we revised our alert system to be based on qSOFA. Similar results were observed in multiple systematic reviews.[11-14] SIRS was found to be more sensitive than qSOFA for the diagnosis of sepsis (risk ratio, 1.32; 95% confidence interval, 0.40-2.24; I^2 = 100%).[11] The pooled specificity of qSOFA in patients with suspected infection outside the ICU was 79.6% (95% confidence interval, 73.3-84.7%).[12] However, current evidence favored qSOFA over SIRS as a predictor of hospital

mortality (risk ratio, 0.03; 95% confidence interval, 0.01-0.05; I^2 = 48%).[11] Therefore, qSOFA may be better than SIRS for identifying high-risk patients.

Table S1: Total number of eligible wards in the participating hospitals**.**

Center	Number of wards
King Abdulaziz Medical City – Riyadh	25 wards
King Abdulaziz Medical City – Jeddah	9 wards
King Abdulaziz Hospital – Al Ahsa	6 wards
Prince Mohammad Bin Abdulaziz Hospital – Madinah	4 wards
Imam Abdulrahman Al Faisal Hospital – Dammam	2 wards
**Total**	**46***

* Two of the wards were combined for purposes of randomization

Table S2: Definitions used in the SCREEN trial.

Item	Description
**Time 0**	Date and time when the patient is checked in for the first time to the ward. If transferred to different wards, take the first date and time only.
**Alert time**	When the patient meets the alert criteria; for patients who have multiple alerts, we will take only the first alert
**Observation time**	The whole hospital stay from time of check-in to the ward to time of discharge from the hospital censored at 90 days. The observation period excludes stays in the operating room, daycare operation room, operation cardiac, outpatient, post-anesthesia care unit, dental outpatient, day surgery, daycare outpatient, intensive care unit, trauma intensive care unit, surgical intensive care unit, Intermediate care unit, progressive care unit, medical cardiac intensive care unit, neurointensive care unit, burn unit).
**Alert population**	Patients who had one alert or more during hospitalization. However, detailed data will be included only for the first alert. For subsequent alerts, only alert count will be provided.

Table S3: Guidelines for nurses and physicians.

GUIDELINES FOR PHYSICIANS

What to do if my patient has an alert?

1- A sign will appear in BESTCare on patient list page when a patient meets the qSOFA criteria "Possible Sepsis Alert" in BESTCare patients' list

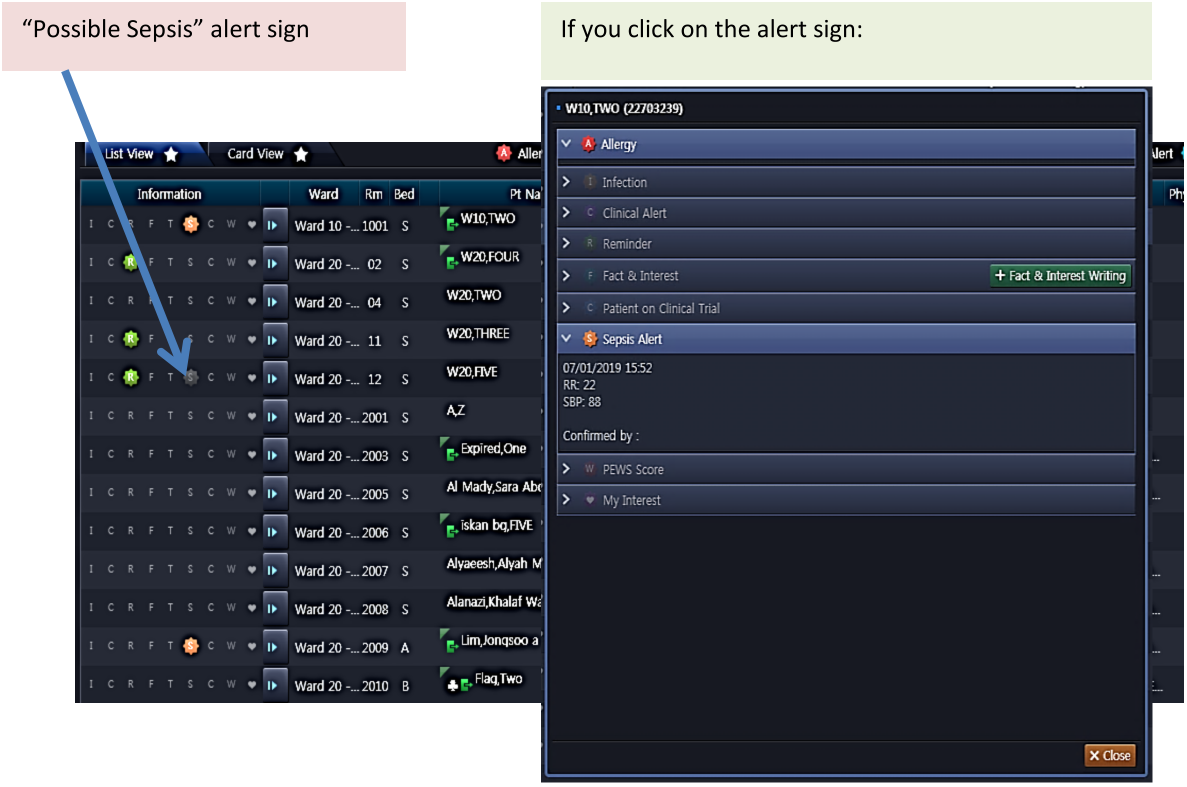

2- A pop-up message will appear in the patient record when patient file is opened. The message asks the physician to assess the patient and document if the patient has sepsis.

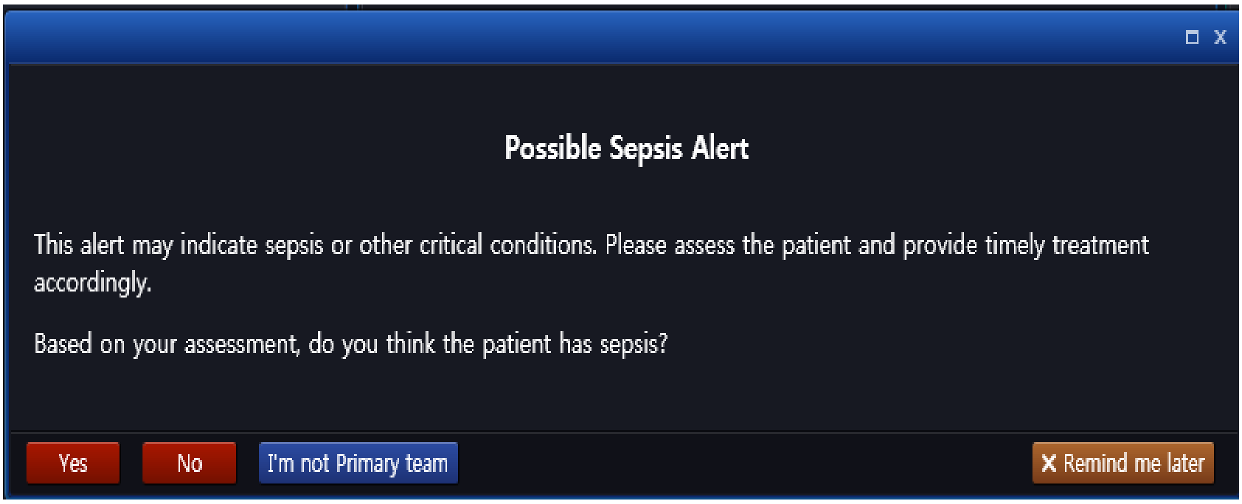

GUIDELINES FOR NURSES

1- Each nurse will have a card that shows who meets qSOFA criteria and when an alert will be activated as shown below.

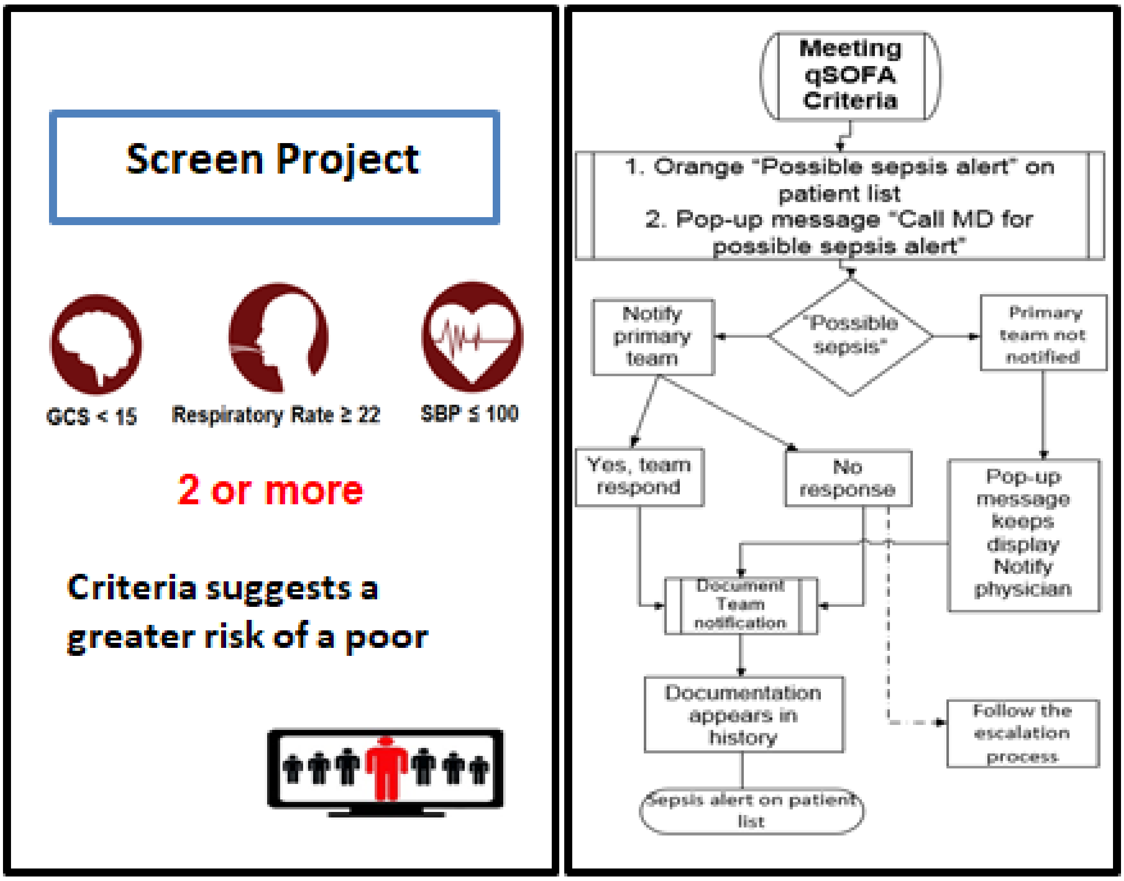

2- The bedside nurse will receive a message when a patient meets qSOFA criteria. The message asks the nurse to immediately inform the primary team and to document that the primary team has been notified. In case of nor response, the nurse will follow an escalation process.

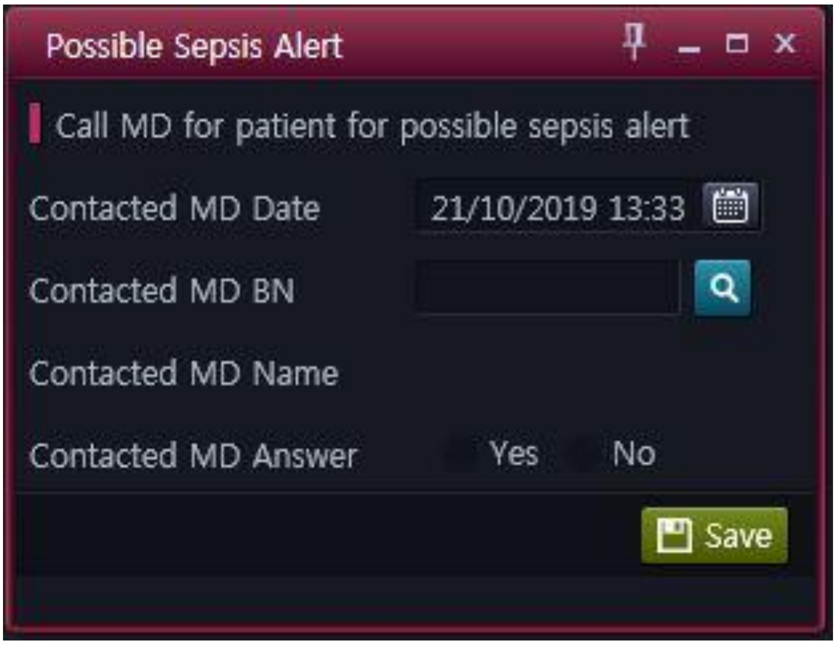

3- The charge nurse will be alerted that the patient met the criteria through an iPod application.

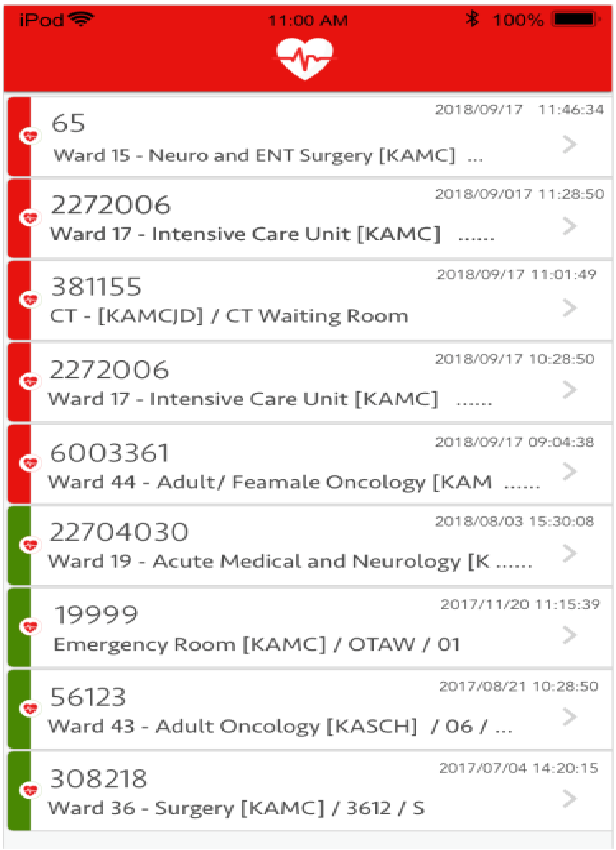

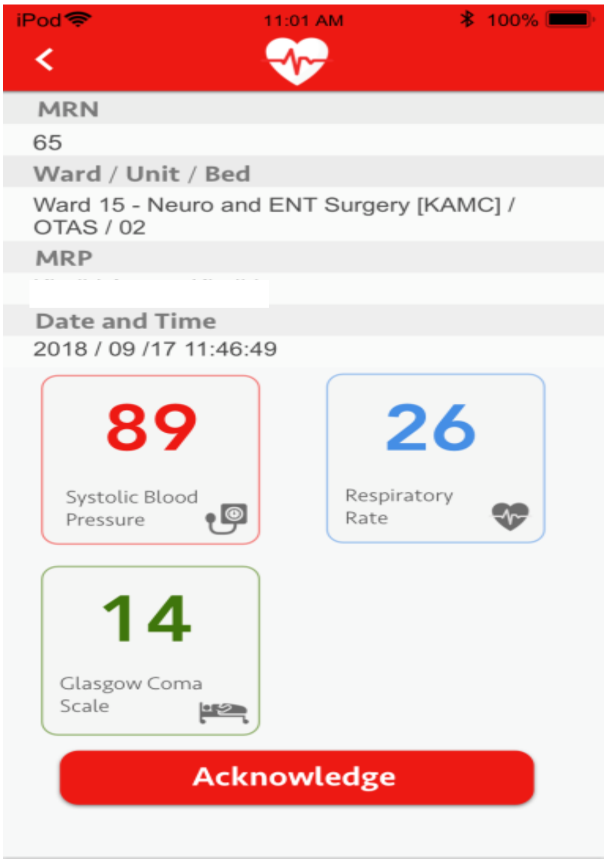

Table S4: The ICD-10-AM codes for comorbid conditions.

Comorbidity	ICD-10-AM Codes
Myocardial infarction	I21.x, I22.x, I25.2
Congestive heart failure	I09.9, I11.0, I13.0, I13.2, I25.5, I42.0, I42.5 - I42.9, I43.x, I50.x, P29.0
Peripheral vascular disease	I70.x, I71.x, I73.1, I73.8, I73.9, I77.1, I79.0, I79.2, K55.1, K55.8, K55.9, Z95.8, Z95.9
Cerebrovascular disease	G45.x, G46.x, H34.0, I60.x - I69.x
Dementia	F00.x - F03.x, F05.1, G30.x, G31.1
Chronic pulmonary disease	I27.8, I27.9, J40.x - J47.x, J60.x - J67.x, J68.4, J70.1, J70.3
Rheumatic disease	M05.x, M06.x, M31.5, M32.x - M34.x, M35.1, M35.3, M36.0
Peptic ulcer disease	K25.x - K28.x
Mild liver disease	B18.x, K70.0 - K70.3, K70.9, K71.3 - K71.5, K71.7, K73.x, K74.x, K76.0, K76.2 - K76.4, K76.8, K76.9, Z94.4
Diabetes without chronic complication	E10.0, E10.1, E10.6, E10.8, E10.9, E11.0, E11.1, E11.6, E11.8, E11.9, E12.0, E12.1, E12.6, E12.8, E12.9, E13.0, E13.1, E13.6, E13.8, E13.9, E14.0, E14.1, E14.6, E14.8, E14.9
Diabetes with chronic complication	E10.2 - E10.5, E10.7, E11.2 - E11.5, E11.7, E12.2 - E12.5, E12.7, E13.2 - E13.5, E13.7, E14.2 - E14.5, E14.7
Hemiplegia or paraplegia	G04.1, G11.4, G80.1, G80.2, G81.x, G82.x, G83.0 - G83.4, G83.9
Renal disease	I12.0, I13.1, N03.2 - N03.7, N05.2 - N05.7, N18.x, N19.x, N25.0, Z49.0 - Z49.2, Z94.0, Z99.2
Any malignancy, including lymphoma and leukemia, except malignant neoplasm of skin	C00.x - C26.x, C30.x - C34.x, C37.x - C41.x, C43.x, C45.x - C58.x, C60.x - C76.x, C81.x - C85.x, C88.x, C90.x - C97.x
Moderate or severe liver disease	I85.0, I85.9, I86.4, I98.2, K70.4, K71.1, K72.1, K72.9, K76.5, K76.6, K76.7
Metastatic solid tumor	C77.x - C80.x
AIDS/HIV	B20.x - B22.x, B24.x

Table S5: The ICD-10-AM codes used for infectious diseases.

Infectious Disease Group	ICD-10-AM Code
Acute lower respiratory tract infections	A481, A482, B59, J09, J100, J101, J108, J110, J111, J118, J120, J121, J122, J128, J129, J13, J14, J150, J151, J152, J153, J154, J155, J156, J157, J158, J159, J160, J168, J170, J171, J172, J173, J178, J180, J181, J182, J188, J189, J200, J201, J202, J203, J204, J205, J206, J207, J208, J209, J210, J218, J219, J22
Breast infections	N61
Central nervous system general infections	G000, G001, G002, G003, G008, G009, G01, G020, G021, G028, G030, G039, G040, G041, G042, G048, G049, G050, G051, G052, G058, G060, G061, G062, G07, G08, G09, G610
Central nervous system viral infections	A801, A802, A803, A804, A809, A811, A812, A818, A819, A820, A821, A829, A830, A831, A832, A833, A834, A835, A836, A838, A839, A840, A841, A848, A849, A850, A851, A852, A858, A86, A870, A871, A872, A878, A879, A880, A881, A888, A89
Connective tissue infectious	M0210, M0211, M0212, M0213, M0214, M0215, M0216, M0217, M0218, M0219, M0230, M0231, M0232, M0233, M0234, M0235, M0236, M0237, M0238, M0239, M0300, M0301, M0302, M0303, M0304, M0305, M0306, M0307, M0308, M0309, M0310, M0311, M0312, M0313, M0314, M0315, M0316, M0317, M0318, M0319, M0320, M0321, M0322, M0323, M0324, M0325, M0326, M0327, M0328, M0329, M0360, M0361, M0362, M0363, M0364, M0365, M0366, M0367, M0368, M0369, M6000, M6001, M6002, M6003, M6004, M6005, M6006, M6007, M6008, M6009, M6300, M6301, M6302, M6303, M6304, M6305, M6306, M6307, M6308, M6309, M6310, M6311, M6312, M6313, M6314, M6315, M6316, M6317, M6318, M6319, M6320, M6321, M6322, M6323, M6324, M6325, M6326, M6327, M6328, M6329, M6500, M6501, M6502, M6503, M6504, M6505, M6506, M6507, M6508, M6509, M6510, M6511, M6512, M6513, M6514, M6515, M6516, M6517, M6518, M6519, M6800, M6801, M6802, M6803, M6804, M6805, M6806, M6807, M6808, M6809, M7100, M7101, M7102, M7103, M7104, M7105, M7106, M7107, M7108, M7109, M7110, M7111, M7112, M7113, M7114, M7115, M7116, M7117, M7118, M7119, M8960, M8961, M8962, M8963, M8964, M8965, M8966, M8967, M8968, M8969
Enteric infections	A000, A001, A009, A010, A011, A012, A013, A014, A020, A021, A022, A028, A029, A030, A031, A032, A033, A038, A039, A040, A041, A042, A043, A044, A045, A046, A047, A048, A049, A050, A051, A052, A053, A054, A058, A059, A060, A061, A062, A063, A064, A065, A066, A067, A068, A069, A070, A071, A072, A073, A078, A079, A080, A081, A082, A083, A084, A085
Enteric symptoms	A090, A099, I880, K528, K529, R11
Gastrointestinal tract infections	K230, K231, K250, K251, K252, K253, K254, K255, K256, K257, K259, K260, K261, K262, K263, K264, K265, K266, K267, K269, K270, K271, K272, K273, K274, K275, K276, K277, K279, K280, K281, K282, K283, K284, K285, K286, K287, K289, K293, K294, K295, K350, K351, K359, K36, K37, K610, K611, K612, K613, K614, K630, K632, K650, K678, K908
Heart and circulatory infections	B332, I00, I010, I011, I012, I018, I019, I020, I029, I050, I051, I052, I058, I059, I060, I061, I062, I068, I069, I070, I071, I072, I078, I079, I080, I081, I082, I083, I088, I089, I090, I091, I092, I098, I099, I301, I330, I339, I38, I390, I391, I392, I393, I394, I398, I400, I410, I411, I412, I430, I716, I790, I791

Infectious Disease Group	ICD-10-AM Code
Hepatic infections	K750, K770, K830
Joint infections	M0000, M0001, M0002, M0003, M0004, M0005, M0006, M0007, M0008, M0009, M0010, M0011, M0012, M0013, M0014, M0015, M0016, M0017, M0018, M0019, M0020, M0021, M0022, M0023, M0024, M0025, M0026, M0027, M0028, M0029, M0080, M0081, M0082, M0083, M0084, M0085, M0086, M0087, M0088, M0089, M0090, M0091, M0092, M0093, M0094, M0095, M0096, M0097, M0098, M0099, M0100, M0101, M0102, M0103, M0104, M0105, M0106, M0107, M0108, M0109, M0110, M0111, M0112, M0113, M0114, M0115, M0116, M0117, M0118, M0119, M0120, M0121, M0122, M0123, M0124, M0125, M0126, M0127, M0128, M0129, M0130, M0131, M0132, M0133, M0134, M0135, M0136, M0137, M0138, M0139, M0140, M0141, M0142, M0143, M0144, M0145, M0146, M0147, M0148, M0149, M0150, M0151, M0152, M0153, M0154, M0155, M0156, M0157, M0158, M0159, M0160, M0161, M0162, M0163, M0164, M0165, M0166, M0167, M0168, M0169, M0180, M0181, M0182, M0183, M0184, M0185, M0186, M0187, M0188, M0189
Kidney infections	N000, N001, N002, N003, N004, N005, N006, N007, N008, N009, N050, N051, N052, N053, N054, N055, N056, N057, N058, N059, N10, N136, N151
Meningococcal disease	A390, A391, A392, A393, A394, A395, A398, A399
Osteomyelitis	M4620, M4621, M4622, M4623, M4624, M4625, M4626, M4627, M4628, M4629, M4630, M4631, M4632, M4633, M4634, M4635, M4636, M4637, M4638, M4639, M4640, M4641, M4642, M4643, M4644, M4645, M4646, M4647, M4648, M4649, M4650, M4651, M4652, M4653, M4654, M4655, M4656, M4657, M4658, M4659
Other bacterial infections	A200, A201, A202, A203, A207, A208, A209, A210, A211, A212, A213, A217, A218, A219, A220, A221, A222, A227, A228, A229, A230, A231, A232, A233, A238, A239, A240, A241, A242, A243, A244, A250, A251, A259, A260, A267, A268, A269, A270, A278, A279, A280, A281, A282, A288, A289, A300, A301, A302, A303, A304, A305, A308, A309, A310, A311, A318, A319, A320, A321, A327, A328, A329, A33, A34, A35, A360, A361, A362, A363, A368, A369, A370, A371, A378, A379, A38, A420, A421, A422, A427, A428, A429, A430, A431, A438, A439, A440, A441, A448, A449, A480, A483, A484, A488, A490, A491, A492, A493, A498, A499, A65, A660, A661, A662, A663, A664, A665, A666, A667, A668, A669, A670, A671, A672, A673, A679, A680, A681, A689, A690, A691, A692, A698, A699, A70, A710, A711, A719, A740, A748, A749, A750, A751, A752, A753, A759, A770, A771, A772, A773, A778, A779, A78, A790, A791, A798, A799, B950, B951, B952, B953, B9541, B9542, B9548, B955, B956, B957, B958, B960, B961, B962, B9631, B9638, B9639, B964, B965, B966, B967, B9681, B9688
Other infectious diseases	B650, B651, B652, B653, B658, B659, B660, B661, B662, B663, B664, B665, B668, B669, B670, B671, B672, B673, B674, B675, B676, B677, B678, B679, B680, B681, B689, B690, B691, B698, B699, B700, B701, B710, B711, B718, B719, B72, B73, B740, B741, B742, B743, B744, B748, B749, B75, B760, B761, B768, B769, B770, B778, B779, B780, B781, B787, B789, B79, B80, B810, B811, B812, B813, B814, B818, B820, B829, B830, B831, B832, B833, B834, B838, B839, B850, B851, B852, B853, B854, B870, B871, B872, B873, B874, B878, B879, B880, B881, B882, B883, B888, B889, B89, B940, B941, B942, B948, B949, B99, E033, E321, F024, F071, I881, I888, I889, T64

Infectious Disease Group	ICD-10-AM Code
Other mycoses	B350, B351, B352, B353, B354, B355, B356, B358, B359, B360, B361, B362, B363, B368, B369, B370, B371, B372, B373, B374, B375, B376, B377, B3781, B3788, B379, B380, B381, B382, B383, B384, B387, B388, B389, B390, B391, B392, B393, B394, B395, B399, B400, B401, B402, B403, B407, B408, B409, B410, B417, B418, B419, B420, B421, B427, B428, B429, B430, B431, B432, B438, B439, B440, B441, B442, B447, B448, B449, B450, B451, B452, B453, B457, B458, B459, B460, B461, B462, B463, B464, B465, B468, B469, B470, B471, B479, B480, B481, B482, B483, B484, B487, B488, B49
Other viral infections	A90, A91, A920, A921, A922, A923, A924, A928, A929, A930, A931, A932, A938, A94, A950, A951, A959, A960, A961, A962, A968, A969, A980, A981, A982, A983, A984, A985, A988, A99, B000, B001, B002, B003, B004, B005, B007, B008, B009, B010, B011, B012, B018, B019, B020, B021, B022, B023, B027, B028, B029, B03, B04, B050, B051, B052, B053, B054, B058, B059, B060, B068, B069, B07, B080, B081, B082, B083, B084, B085, B088, B09, B250, B251, B252, B258, B259, B260, B261, B262, B263, B268, B269, B270, B271, B278, B279, B331, B333, B334, B338, B340, B341, B342, B343, B344, B348, B349, B970, B971, B972, B973, B974, B975, B976, B977, B978
Postoperative infections	T802, T8141, T8142, T826, T827, T835, T836, T845, T846, T847, T8571, T8572, T8578, T874
Reproductive system infections, female	N700, N701, N709, N710, N711, N719, N72, N730, N731, N732, N733, N734, N735, N736, N738, N739, N748, N751, N764, N870, N871, N872, N879
Reproductive system infections, male	N410, N411, N412, N413, N431, N450, N459, N481, N482, N490, N491, N492, N498, N499, N510, N511, N512, N518
Septicaemia	A400, A401, A402, A403, A408, A409, A410, A411, A412, A413, A414, A4150, A4151, A4152, A4158, A418, A419
Skin infections, other	B86, S1013, S1083, S1093, S2013, S2033, S2043, S2083, S3083, S3093, S4083, S5083, S6083, S7083, S8083, S9083, T009, T0903, T1108, T1303, T1403, T633, T634, T793, T8901, T8902
Skin infections, typical	A46, L00, L010, L011, L020, L021, L022, L023, L024, L028, L029, L0301, L0302, L0310, L0311, L032, L033, L038, L039, L040, L041, L042, L043, L048, L049, L050, L080, L081, L088, L089
Tuberculosis	A150, A151, A152, A153, A154, A155, A156, A157, A158, A159, A160, A161, A162, A163, A164, A165, A167, A168, A169, A170, A171, A178, A179, A180, A181, A182, A183, A184, A185, A186, A187, A188, A190, A191, A192, A198, A199, J65, N740, N741
Upper respiratory tract infections	J00, J010, J011, J012, J013, J014, J018, J019, J020, J028, J029, J030, J038, J039, J040, J041, J042, J050, J051, J060, J068, J069, J320, J321, J322, J323, J324, J328, J329, J340, J36, J370, J371, J390, J391
Urinary tract infections	N300, N341, N351, N370, N378, N390

Table S6: List of antibiotics based on the hospital formulary.

**Antibiotics**
Aztreonam
Azithromycin
Piperacillin/Tazobactam
Vancomycin
Ceftriaxone
Metronidazole
Imipenem/Cilastin
Moxifloxacin
Gentamicin
Meropenem
Clindamycin
Metronidazole
Cefepime
Caspofungin
Fluconazole
Linezolid
Cloxacillin
Amikacin
Cefazolin
Cefuroxime
Colistin
Ciprofloxacin
Ticarcillin/Clavulanate
Ampicillin/Sulbactam
Amoxicillin
Ceftazidime
Ceftazidime/avibactam
Tigecycline
Trimethoprim-sulfamethoxazole
Amphotericin-B
Voriconazole
Anidulafungin
Doxyclycline

Table S7: Baseline data.

Variable	Definitions
Age (yr), Median (Q1, Q3)	Age
Sex (number, %)	Gender
Admission source (number, %)	location of the patient before ward check-in
Emergency Room	Emergency department
Operating Room	Operative room
Clinic	Outpatient
Intensive care unit	Intensive care unit (cardiac and non-cardiac)
Others	Any other which may include: other hospitals, other ward
**Service (number, %)**	
Medical	Medical wards include: adult diabetic and endocrine center, Adult Hepatology, Endocrinology & Metabolism; Gastroenterology; Hepatobiliary Sciences; Internal Medicine; Nephrology; Neurology; Palliative Care; Psychiatry; Pulmonology; Rheumatology
Surgical	Surgical wards include: adult organ transplant & hepatobiliary surgery, Adult Transplant Nephrology; Anesthesiology, Breast Surgery; General Surgery; Neurosurgery; Oral & Maxillofacial Surgery; Orthopedics; Podiatric Surgery; Plastic Surgery; Thoracic Surgery; Urology Surgery; Vascular Surgery; ENT
Hematology-Oncology	Hematology-oncology wards include: Adult hematology; Adult medical oncology; Adult hematology and oncology; Adult Stem Cell Transplant & Cellular Therapy; Radiation Oncology
Other	Intensive Care - Adult; Obstetrics & Gynecology; Ophthalmology
**Admitting ward (number, %)**	
Medical	Riyadh: ward 7, ward 8, ward 10, ward 12, ward 13, ward 19, ward 20, ward 22, ward 23, ward 24, ward 25, AMU<br>Jeddah: ward 4, ward 5, ward 6<br>Madinah: ward 8<br>Dammam: none<br>Al Ahsa : ward 3, ward 7
Surgical	Riyadh: ward 15, ward 18, ward 36, ward 37, ward 38, ward 39, ward 40, ASU<br>Jeddah: ward 14, ward 16<br>Madinah: ward 5<br>Dammam: none<br>Al Ahsa: ward 2, ward 8
Oncology	Riyadh: ward 41, ward 43, ward 44<br>Jeddah: ward 22, ward 23, ward 25<br>Madinah: none<br>Dammam: none<br>Al Ahsa : ward 5
Mixed (any combination)	Riyadh: ward 16 A, B, C, ward 49<br>Jeddah: ward 3<br>Madinah: ward 3, ward 6<br>Dammam: ward 1, ward 3<br>Al Ahsa: ward 6
**Admission diagnosis, main and secondary**	Admission note, problem list, or ICD-10-AM code

**Comorbidities (number, %)**	
End-Stage Renal Disease (ESRD)	ICD-10-AM *
Chronic liver disease	
Cancer without metastasis	
immune-compromised	
Diabetes non-complicated	
Diabetes, complicated	
Congestive heart failure	
Acquired immunodeficiency syndrome	
Moderate to severe Chronic Kidney Disease (CKD)	
Myocardial infarction	
Chronic pulmonary disease	
Peripheral vascular disease	
Stroke or transient ischemic attack	
Dementia	
Hemiplegia or paraplegia	
Connective tissue disease	
Peptic ulcer disease	
Mild liver disease	
Moderate to severe liver disease	
Charlson Comorbidity Index	
**Source of infection on admission (number, %)**	
No infection	
Pneumonia	
Urinary tract infection	
Skin and soft tissue infection	
Intra-abdominal infection	
other infections	
**Dialysis**	
Alert count	
Time to first alert	
Alert information criteria leading to triggering (number, %)	
RR≥22 (breath/minute)	Respiratory Rate
SBP≤100 mm Hg	Systolic Blood Pressure
GCS<15	Glasgow Coma Scale

Table S8: Physiological parameters and treatments at baseline and pre-alert.

Variable	Description
**Vital Signs**	
**Blood Pressure**	
Baseline systolic blood pressure	The lowest value in the first 12 hours of check in to the ward
Baseline diastolic blood pressure	The lowest value in the first 12 hours of check in to the ward
Pre-alert systolic blood pressure	The lowest value in the 12 hours before the alert
Pre-alert diastolic blood pressure	The lowest value in the 12 hours before the alert
**Heart Rate**	
Baseline heart rate	The highest value in the first 12 hours of check in to the ward
Pre-alert heart rate	The highest value in the 12 hours before the alert
**Temperature**	
Baseline temperature	The highest value and lowest value in the first 12 hours of check in to the ward
Pre-alert temperature	The highest value and lowest value in the 12 hours before the alert
**Respiratory Rate**	
Baseline respiratory rate	The highest value in the first 12 hours of check in to the ward
Pre-alert respiratory rate	The highest value in the 12 hours before the alert
**Lab parameters**	
**Lactate**	
Baseline lactate (mmol/L)	The highest value 12 hours before check in to the ward to 12 hours of check-in to the ward
Pre-alert lactate (mmol/L)	The highest value in the 12 hours before the alert.
**Complete Blood Count**	
Baseline white blood cells 10^9/L	The highest value in the 12 hours before check-in to the ward to 12 hours after check-in to the ward
Pre-alert white blood cells 10^9/L	The highest value in the 12 hours before the alert
**Bilirubin**	
Baseline bilirubin (µmol/L)	The highest value in the 12 hours before check-in to the ward to 12 hours after check-in to the ward
Pre-alert bilirubin (µmol/L)	The highest value in the 12 hours before the alert
**Creatinine**	
Baseline creatinine (µmol/L)	The highest value in the 12 hours before check-in to the ward to 12 hours after check-in to the ward
Pre-alert creatinine µmol/L)	The highest value in the 12 hours before the alert
**Culture (number, %)**	
Baseline blood culture	Blood culture ordered in the 12 hours before check-in to the ward to 12 hours after check-in to the ward, results
Pre-alert blood culture	Blood culture ordered in the 12 hours before the alert
Baseline respiratory culture	Respiratory culture ordered in the 12 hours before check-in to the ward to 12 hours after check-in to the ward (number, %), results
Pre-alert respiratory culture	Respiratory culture ordered in the 12 hours before the alert
Baseline urine culture	Urine culture ordered in the 12 hours before check-in to the ward to 12 hours after check-in to the ward, results
Pre-alert urine culture	Urine culture ordered in the 12 hours before alert
Baseline Body fluid Culture (pleural, ascitic, CSF, joint)	Body fluid culture ordered in the 12 hours before check-in to the ward to 12 hours after check-in to the ward, results
Pre-alert Body fluid culture	Body fluid culture ordered in the 12 hours before alert
**Intravenous fluids (number, %)**	
Baseline Intravenous fluids (IV)	Order of IV fluids (NS, ½NS, D5NS, D5½NS, LR, D5WLR, Albumin 5%, 20%) in the 12 hours before check-in to the ward to 12 hours after check-in to the ward
Pre-alert IVF	Order of IV fluids (NS, ½NS, D5NS, D5½NS, LR, D5WLR, Albumin 5%, 20%) in the 12 hours before the alert
**Antibiotics (number, %)**	
Baseline Antibiotics	antibiotics ordered in the 12 hours before check-in to the ward to 12 hours after check-in to the ward
Pre-alert Antibiotics	antibiotics ordered in the 12 hours before alert

Table S9: Process Measures and post-alert physiologic variables.

Variable	Description
Post-alert lactate	Reported in the 12 hours after the alert (number, %)<br>Highest value reported in the 12 hours after the alert
Post- alert blood culture (number, %)	Blood culture ordered in the 12 hours after alert
Post-alert respiratory culture (number, %)	Respiratory culture ordered in the 12 hours after alert
Post-alert urine culture (number, %)	Urine culture ordered in the 12 hours after alert
Post-alert body fluid culture (number, %)	Body fluid culture ordered in the 12 hours after alert
Antibiotics (number, %)	New antibiotics administered in the 12 hours after the alert<br>New antibiotics administered in the 3 hours after the alert
Post-alert systolic blood pressure	The lowest value in the 12 hours after the alert
Post-alert diastolic blood pressure	The lowest value in the 12 hours after the alert
Post-alert heart rate	The highest value in the 12 hours after the alert
Post-alert respiratory rate (RR)	Highest value RR in the 12 hours after the alert

Table S10: Outcomes.

Variable	Description
In-hospital mortality (number, %)	Percentage of patients who die in the hospital up to 90 days
Hospital length of stay	Stay in the hospital in days censored at 90 days
ICU admission	Admission to ICU anytime during this hospital admission from check-in* to the ward up to 90 days; date and time, number of days in ICU
Post-alert ICU admission	Transfer to ICU within 14 days of the alert (number, %)
ICU-free days	Number of days during the measurement period (maximum of 90 days) that the patient is both alive and free of mechanical ventilation
Incident renal replacement therapy (number, %)	Renal replacement therapy (hemodialysis or continuous renal replacement therapy) any time this hospital admission after check-in to the ward up to 90 days
Post-alert renal replacement therapy (RRT) (number, %)	Renal replacement therapy within 14 days from the alert
Vasopressor therapy (number, %)	Vasopressors (norepinephrine, dopamine, epinephrine, dobutamine, phenylephrine, vasopressin) within 90 days of check-in; date and time of initiation
Post-alert vasopressors (number, %)	Vasopressors within 14 days of the alert; date and time of initiation
Mechanical ventilation (number, %)	Mechanical ventilation (except in the operating room) anytime this hospital admission after check-in to the ward to 90 days
Post-alert Mechanical ventilation (number, %)	Mechanical ventilation (except in the operating room) within 14 days of the alert
Critical Care Rapid Response Team (CCRT) activation (number, %)	CCRT activation during this hospital admission after check-in to the ward; date and time
Post-alert CCRT activation (number, %)	CCRT activation within 14 days of the alert; date and time
Code Blue (number, %)	Code Blue activation during this hospital admission after check-in to the ward (number, %) Date and time
Post-alert Code Blue (number, %)	Code Blue activation within 14 days of the alert; date and time
Post-alert antibiotics (number, %)	Antibiotic ordered within 14 days of the alert
Antibiotic free-days up to 90 days	Number of days during the measurement period (maximum of 90 days) that the patient is both alive and free of antibiotics
Multidrug-resistant organism (number, %)	A positive culture for multidrug-resistant Acinetobacter, Pseudomonas, bacteria with extended-spectrum beta-lactamases, methicillin-resistant Staphylococcus aureus, vancomycin-resistant enterococcus, carbapenem-resistant Klebsiella pneumoniae during 90 days from check-in
Post-alert MDROs (number, %)	Starting from alert, positive culture for multidrug-resistant Acinetobacter, Pseudomonas, bacteria with extended-spectrum beta-lactamases, methicillin-resistant Staphylococcus aureus, vancomycin-resistant enterococcus, carbapenem-resistant Klebsiella pneumoniae up to 90 days
Clostridium difficile infection (number, %)	Positive Clostridium difficile toxins by serology or polymerase chain reaction during 90 days from check-in
Alert Clostridium difficile infection (number, %)	Start from alert: Positive Clostridium difficile toxins by serology or polymerase chain reaction during 90 days

*Check in to ward: ward admission; ward check out: ward discharge

Table S11: Justification for Sample Size calculation.

Table S11 A: A retrospective historical electronic data from 01July 2018 to 30 June 2019 was used to consider inputs for sample size calculation. The summary of the results are as follow.

Table of Alert by deaths			
	Death		
**Alert (qSofa Alert)**	**Y**	**N**	**Total**
**Y**	**565** (8.16)	**6356** (91.84)	**6921 (**18.28)
**N**	**620** (2.00)	**30311** (98.00)	**30931 (**81.72)
**Total**	**1185** (3.13)	**36667** (96.87)	**37852 (**100.00)

Table S11 B: Assumptions used for the sample size calculation.

	**Inputs**	**Justification**
A) The implementation of qSOFA sepsis alert affects only patients with qSOFA sepsis alert	Yes	i.e. 6921 patients
B) Only half of patients with qSOFA sepsis alert have sepsis	Yes	6921/2= 3461
C) 90% of deaths in the sepsis alert group are sepsis related	Yes	Sepsis-related mortality=565*0.9=509<br>Non-sepsis related mortality=56
D) Early intervention resulting from qSOFA sepsis alert will reduce the sepsis-related mortality 50%	8.16% to 4.08%	Sepsis related mortality 509 deaths will be reduced by 50% to 255.
• Expected mortality rate before intervention is:	3.13%	A total of 1185 deaths out of 37852 patients.
• Mortality rate expected to be reduced after intervention :	2.46%	620+255+56 deaths= 931<br><br>Then mortality rate =931/37852=2.46
• Difference in mortality rate	0.67%	Reduction in mortality after intervention
• Relative risk	0.79	
D) Expected intra-cluster correlation	0.22	
Study Power	80%	
Level of significance	5%	
Sample Size	65250	An average of 1450 subjects per cluster with an average of 145 subjects per cluster per time period.

qsofa: quick Sequential Organ Failure Assessment

References

1. Arabi YM, Al-Dorzi HM, Alamry A, Hijazi R, Alsolamy S, Al Salamah M, Tamim HM, Al-Qahtani S, Al-Dawood A, Marini AM *et al*: **The impact of a multifaceted intervention including sepsis electronic alert system and sepsis response team on the outcomes of patients with sepsis and septic shock**. *Ann Intensive Care* 2017, **7**(1):57.
2. Manaktala S, Claypool SR: **Evaluating the impact of a computerized surveillance algorithm and decision support system on sepsis mortality**. *Journal of the American Medical Informatics Association* 2016, **24**(1):88-95.
3. Sawyer AM, Deal EN, Labelle AJ, Witt C, Thiel SW, Heard K, Reichley RM, Micek ST, Kollef MH: **Implementation of a real-time computerized sepsis alert in nonintensive care unit patients**. *Critical care medicine* 2011, **39**(3):469-473.
4. Mdege ND, Man MS, Taylor Nee Brown CA, Torgerson DJ: **Systematic review of stepped wedge cluster randomized trials shows that design is particularly used to evaluate interventions during routine implementation**. *J Clin Epidemiol* 2011, **64**(9):936-948.
5. Porsdam Mann S, Savulescu J, Sahakian BJ: **Facilitating the ethical use of health data for the benefit of society: electronic health records, consent and the duty of easy rescue**. *Philosophical transactions Series A, Mathematical, physical, and engineering sciences* 2016, **374**(2083).
6. Gonzales R, Anderer T, McCulloch CE, Maselli JH, Bloom FJ, Graf TR, Stahl M, Yefko M, Molecavage J, Metlay JP: **A Cluster-Randomized Trial of Decision Support Strategies for Reducing Antibiotic Use for Acute Bronchitis**. *JAMA internal medicine* 2013, **173**(4):267-273.
7. van Wyk JT, van Wijk MA, Sturkenboom MC, Mosseveld M, Moorman PW, van der Lei J: **Electronic alerts versus on-demand decision support to improve dyslipidemia treatment: a cluster randomized controlled trial**. *Circulation* 2008, **117**(3):371-378.
8. Dykes LA, Heintz SJ, Heintz BH, Livorsi DJ, Egge JA, Lund BC: **Contrasting qSOFA and SIRS Criteria for Early Sepsis Identification in a Veteran Population**. *Federal Practitioner* 2019, **36**(Suppl 2):S21.
9. Anand V, Zhang Z, Kadri SS, Klompas M, Rhee C, Program CPE: **Epidemiology of Quick Sequential Organ Failure Assessment Criteria in Undifferentiated Patients and Association With Suspected Infection and Sepsis**. *Chest* 2019.
10. Arabi Y, Al-Hameed F, Alyafi W, AlQarni A, Shouabi AA, AlZahrani M, Karsou S, Yami A, Jastaniah W, Wazzan A *et al*: **1574: QSOFA COMPARED TO SIRS IN PREDICTING MORTALITY AMONG WARD PATIENTS IN 5 HOSPITALS**. *Critical Care Medicine* 2019, **47**(1):762.
11. Serafim R, Gomes J, Salluh J, Póvoa P: **A Comparison of the Quick-SOFA and Systemic Inflammatory Response Syndrome Criteria for the Diagnosis of Sepsis and Prediction of Mortality: A Systematic Review and Meta-Analysis**. In.; 2018.
12. Fernando S, Tran A, Taljaard M, Cheng W, Rochwerg B, Seely A, Perry J: **Prognostic Accuracy of the Quick Sequential Organ Failure Assessment for Mortality in Patients With Suspected Infection: A Systematic Review and Meta-analysis**. In.; 2018.
13. Waligora G, Gaddis G, Church A, Mills L: **Rapid Systematic Review: The Appropriate Use of Quick Sequential Organ Failure Assessment (qSOFA) in the Emergency Department**. *The Journal of emergency medicine*:S0736-4679 (0720) 30594-30591.
14. Gando S, Shiraishi A, Abe T, Kushimoto S, Mayumi T, Fujishima S, Hagiwara A, Shiino Y, Shiraishi S-i, Hifumi T: **The SIRS criteria have better performance for predicting infection than qSOFA scores in the emergency department**. *Scientific Reports* 2020, **10**.
